# Effects of *Zanthoxylum bungeanum* Leaves on Production Performance, Egg Quality, Antioxidant Status, and Gut Health in Laying Hens

**DOI:** 10.3390/ani16020273

**Published:** 2026-01-16

**Authors:** Qiaobo Lei, Xinglai Li, Shanchuan Cao, Jianfei Zhao, Jingbo Liu

**Affiliations:** 1 College of Life Sciences and Agri-Forestry, Southwest University of Science and Technology, Mianyang 621010, China; qiaobo100@163.com (Q.L.); lixinglai2023@163.com (X.L.); shanchuancao@swust.edu.cn (S.C.); zhaojf@swust.edu.cn (J.Z.); 2 Tongwei Agricultural Development Co., Ltd., Chengdu 610096, China

**Keywords:** *Zanthoxylum bungeanum* leaves, production performance, egg quality, intestinal health, cecal microbiota, laying hens

## Abstract

*Zanthoxylum bungeanum* leaves (ZBL) are a phytogenic feed resource, but their energy value and functional effects in laying hens are not well defined. Two experiments were conducted. In Exp. 1, 96 healthy 38-week-old Roman Pink laying hens were allotted to either a control diet or a diet containing 5% ZBL (eight replicates, six hens per replicate) to determine apparent metabolizable energy (AME) using an indicator method (7 d adaptation, 3 d collection). The AME and nitrogen-corrected AME of ZBL were 5.46 and 5.33 MJ/kg, respectively. In Exp. 2, 832 healthy 41-week-old hens were randomly assigned to diets supplemented with 0, 1%, 2%, or 3% ZBL (8 replicates, 26 hens per replicate) for 8 weeks after 1 week adaptation. Dietary ZBL at 1% to 3% did not affect production performance (*p* > 0.05), but increased albumen height linearly (*p* < 0.05) and improved yolk color at 2% and 3% (*p* < 0.05). ZBL increased serum albumin (*p* < 0.05) with a linear tendency (*p* = 0.065), and elevated serum IgA and IgM linearly (*p* < 0.05). Serum total antioxidant capacity and total superoxide dismutase were increased (*p* < 0.05) with significant linear and quadratic responses (*p* < 0.05), while serum malondialdehyde was reduced (*p* < 0.05). In the liver, 3% ZBL increased total antioxidant capacity (*p* < 0.05), hepatic catalase activity was decreased in all ZBL groups (*p* < 0.05), and hepatic malondialdehyde was reduced (*p* < 0.05). Cecal acetate increased linearly (*p* < 0.05), and propionate and butyrate increased with both linear and quadratic dose responses (*p* < 0.05). ZBL improved small intestinal morphology, especially duodenal villus height (*p* < 0.05). Gut microbiota was remodeled, with a marked reduction in *norank_o__WCHB1-41* and increases in *Ruminococcus*, *Pseudoflavonifractor*, and several *Coriobacteriales* and *Erysipelatoclostridiaceae* taxa. Overall, ZBL provides usable energy and, at 2–3% inclusion, enhances egg quality, antioxidant status, humoral immunity, short-chain-fatty-acid production, and intestinal health without compromising laying performance.

## Simple Summary

Laying hens need good quality feed to produce safe, nutritious eggs, but conventional feeds are becoming more expensive and compete with human food. At the same time, large amounts of *Zanthoxylum bungeanum* leaves (ZBL) are discarded each year, which come from *Zanthoxylum bungeanum*, whose fruits are widely used as a pungent spice in Chinese cuisine. In this study, we first measured how much usable energy ZBL can provide for hens. Diets containing different levels of ZBL were then fed to laying hens, and the following were monitored: egg production, egg quality, blood health markers, the ability to resist oxidative damage, gut structure, and gut bacteria. We found that ZBL provided relatively low energy but did not harm egg production when it replaced part of a low-energy-feed ingredient. In contrast, it improved egg white and yolk quality, boosted the antioxidant and immune status of laying hens, improved the structure of the small intestine, and increased beneficial fermentation products in the cecum. These results suggest that using ZBL in layer diets can turn agricultural waste into a valuable feed resource that supports both egg quality and hens’ health.

## 1. Introduction

Eggs are widely consumed worldwide, and the profitability of layer production is increasingly constrained by high feed costs and restrictions on the use of in-feed antibiotics. Plant-derived additives are therefore being explored as natural tools to support performance, egg quality, and health. Meta-analytic and experimental studies suggest that phytogenic extracts and Chinese herbal mixtures can improve laying rate, egg mass, and feed efficiency, while enhancing antioxidant and immune status in hens without compromising egg quality [1,2].

*Zanthoxylum bungeanum* Maxim is an important woody economic crop in China. Its pericarp is widely used as a culinary spice and in traditional Chinese medicine, while the large quantity of leaves produced during cultivation and processing is often discarded and remains underutilized. Phytochemical analyses have shown that the leaves are rich in flavonoids such as quercetin and its glycosides, apigenin glycosides, and other flavonoid compounds, as well as polyphenols, essential oils, and related bioactive constituents, and display marked free radical scavenging and antioxidant capacities [3,4]. In addition, polyphenols from *Z. bungeanum* have been demonstrated to exert anti-inflammatory and immunomodulatory activities and show promising potential in the prevention and management of inflammation-related diseases [5]. In laying hens, a recent study reported that dietary *Z. bungeanum* leaf (ZBL) can enhance yolk color and flavor without depressing laying rate, accompanied by shifts in yolk lipid composition [6]. In broilers, the inclusion of ZBL powder at moderate levels has been reported to improve growth performance and antioxidant capacity and to support intestinal barrier function, partly through the modulation of the Nrf2 and HO-1 pathway [7]. Seed meal and other *Z. bungeanum* coproducts have also shown promise as unconventional protein or energy ingredients in poultry diets [8]. However, information on the metabolizable energy value of ZBL for layers, the optimal inclusion level, and its broader effects on systemic metabolism, intestinal morphology, cecal fermentation, and microbiota remains scarce.

Therefore, the present study aimed to evaluate the effects of ZBL on laying performance, egg quality, and key indicators of health in laying hens. By clarifying the nutritional value and functional effects of ZBL in practical diets, this work seeks to provide a scientific basis for their use as a new feed resource in the egg industry, with the potential to reduce feed cost, increase the added value of orchard byproducts, alleviate pressure on conventional grain and protein ingredients, and support more sustainable layer production.

## 2. Materials and Methods

### 2.1. Preparation and Nutrient Composition of ZBL

Fresh ZBL was dried in a forced-air oven and subsequently milled. For chemical analyses, the ground samples were prepared using different sieve sizes according to the requirements of the corresponding analytical standards. Specifically, samples intended for proximate composition analyses were ground to pass through a 0.42 mm sieve, in accordance with the Chinese National Standard GB/T 20195-2024 [9]. For crude fiber determination, samples were ground to pass through a 1.0 mm sieve, as required by the relevant method standard. Representative subsamples were then used to determine proximate composition and other nutritional indices, and the analyzed nutrient composition of ZBL is presented in Table 1. Dry matter content of ZBL was determined by drying samples in a hot air oven at 135 °C for 2 h according to GB/T 6435-2014 [10]. Crude protein (CP) was analyzed following GB/T 6432-2018 [11] using an elemental analyzer (CHNS 932; Leco Corporation, St. Joseph, MI, USA). Crude fat was determined by ether extraction in accordance with GB/T 6433-2006 [12] using a Soxhlet-type fat extractor (B-811, Büchi Labortechnik AG, Flawil, Switzerland). Crude fiber was measured according to GB/T 6434-2022 [13] with a fiber analyzer (ANKOM 2000i, ANKOM Technology, Macedon, NY, USA), and crude ash was determined by incineration in a muffle furnace (SX2-4-10N, Shanghai Yiheng Scientific Instruments Co., Shanghai, China) following GB/T 6438-2007 [14]. Calcium and total phosphorus contents were analyzed according to GB/T 6436-2018 [15] and GB/T 6437-2018 [16], respectively, after wet digestion, using a UV–visible spectrophotometer (UV-2450, Shimadzu Corporation, Kyoto, Japan). Gross energy was measured by adiabatic bomb calorimetry in accordance with ISO 9831:1998 [17] using a bomb calorimeter (6400, Parr Instrument Company, Moline, IL, USA). Total polyphenol content was quantified using a colourimetric assay based on T/AHFIA 005-2018 [18] with the same UV–visible spectrophotometer, and total flavonoid content was determined following the procedures described in the Technical Guidelines for Testing and Evaluation of Physicochemical and Hygienic Indices of Health Food (2020 edition) [19].

### 2.2. Experiment 1: Determination of Apparent Metabolizable Energy of ZBL

#### 2.2.1. Experimental Design and Management

A metabolism trial was conducted to determine the apparent metabolizable energy (AME) and nitrogen-corrected AME (AMEn) of ZBL for laying hens. Ninety-six healthy Lohmann Pink laying hens at thirty-eight weeks of age with similar body weight were randomly allocated to two dietary treatments, with eight replicates per treatment and six hens per replicate (one hen per cage, six adjacent cages constituting one replicate). The control group was fed a corn and soybean meal-based basal diet, and the test group received a diet in which 5% ZBL was added to the basal diet. The basal diet was formulated according to the NRC (1994) [20] and the Chinese Feeding Standard of Chicken (NY/T 33-2004) [21], and its ingredient composition and nutrient levels are shown in Table 2. All diets were provided in mash form, and ZBL was incorporated into the basal diet by stepwise mixing. Chromium oxide (Cr_2_O_3_) was included in both diets at 0.5% as an indigestible marker. The experiment consisted of a 7 d adaptation period followed by a 3 d collection period. The trial was conducted at the experimental station of the Institute of Animal Nutrition, Sichuan Agricultural University. During the experiment, room temperature was maintained at 20–25 °C. Hens were fed twice daily at approximately 09:00 and 15:00, and a 16 h light and 8 h dark lighting program was applied. All birds had free access to feed and water, and cages from different treatments were distributed evenly within the house to minimize environmental variation.

#### 2.2.2. Excreta Collection and Sample Preparation

During the 3 d collection period, excreta were collected every 12 h from each replicate. Feathers, feed residues, and other foreign materials were removed manually. After each collection, excreta were pooled by replicate, acidified with 10% dilute sulfuric acid to fix nitrogen, and stored at −20 °C. At the end of the collection period, the pooled excreta of each replicate were thoroughly mixed, dried in a forced-air oven at 65 °C for 72 h, and then allowed to equilibrate at room temperature for 24 h. The dried samples were ground and passed through a 40-mesh sieve and stored for subsequent analyses.

#### 2.2.3. Determination of Chromium and Calculation of AME and AMEn

Chromium concentrations in diets and excreta were determined by wet digestion and spectrophotometry. Briefly, an appropriate amount of sample was placed in a 100 mL beaker with 30 mL concentrated nitric acid and heated to boiling for approximately 45 min. Then, 9 mL of 70% perchloric acid was added, and heating was continued until the solution became dark red. After cooling, the digest was transferred quantitatively to a 100 mL volumetric flask, brought to volume with distilled water, and mixed thoroughly. Chromium concentration was measured at 440 nm using a spectrophotometer. The gross energy (GE) of diets and excreta was determined by bomb calorimetry. Using chromium as an indigestible marker, the apparent metabolizability coefficient of dietary energy was calculated by the indicator ratio method:

$$\mathrm{Energy}\;\mathrm{metabolizability}\;\mathrm{in}\;\mathrm{diet}\;(\%)=100-100\times \left(\frac{\mathrm{marker}\;\mathrm{in}\;\mathrm{diet}}{\mathrm{marker}\;\mathrm{in}\;\mathrm{excreta}}\right)\times \left(\frac{\mathrm{GE}\;\mathrm{in}\;\mathrm{excreta}}{\mathrm{GE}\;\mathrm{in}\;\mathrm{diet}}\right).$$

The apparent metabolizable energy (AME, MJ/kg) of each diet was then calculated as:

$$\mathrm{AME}\;\mathrm{of}\;\mathrm{diet}\;(\mathrm{MJ}/\mathrm{kg})=\mathrm{GE}\;\mathrm{of}\;\mathrm{diet}\;(\mathrm{MJ}/\mathrm{kg})\times \frac{\mathrm{Energy}\;\mathrm{metabolizability}\;\mathrm{in}\;\mathrm{diet}\;(\%)}{100}$$

The AME of ZBL (MJ/kg) was calculated using the conventional difference method:

$$\mathrm{AME}\;\mathrm{of}\;\mathrm{ZBL}\;(\mathrm{MJ}/\mathrm{kg})=\frac{\mathrm{ME}\;\mathrm{of}\;\mathrm{test}\;\mathrm{diet}\;(\mathrm{MJ}/\mathrm{kg})-(1-i)\times \mathrm{ME}\;\mathrm{of}\;\mathrm{basal}\;\mathrm{diet}\;(\mathrm{MJ}/\mathrm{kg})}{i}$$

where i is the inclusion rate of ZBL in the test diet.

Nitrogen-corrected AME of ZBL (AMEn, MJ/kg) was calculated as:

AMEn of ZBL (MJ/kg)=AME of ZBL (MJ/kg)−(RN×0.03439)

where RN is nitrogen retained (g/kg feed intake). The constant 34.39 kJ/g for urinary nitrogen was converted to MJ/g as 0.03439 MJ/g. RN was estimated as:

$$\mathrm{RN}\;(g/\mathrm{kg})=\frac{\mathrm{crude}\;\mathrm{protein}\;\mathrm{content}\;(g/\mathrm{kg})}{6.25},$$

with 6.25 as the conversion factor from nitrogen to crude protein.

### 2.3. Experiment 2: Effects of Different Dietary Levels of ZBL on Laying Performance, Egg Quality, and Intestinal Health in Laying Hens

#### 2.3.1. Experimental Design, Diets, and Management

A total of 832 healthy Lohmann Pink laying hens at 41 weeks of age with similar body weight and laying rate were used in this experiment. The hens were randomly assigned to 4 dietary treatments, with 8 replicate groups per treatment and 26 hens in each replicate. The 4 treatments consisted of a basal diet supplemented with 0 (CON), 1% (ZBL1), 2% (ZBL2), or 3% (ZBL3) ZBL powder. The basal diet was formulated to meet the nutrient requirements of laying hens according to current recommendations. ZBL were incorporated into the basal diet by partially replacing corn and soybean meal (Table 3). All diets were produced in mash form and mixed in a commercial feed mill to ensure uniformity. The hens were allowed a one-week adaptation period to the experimental diets, followed by an eight-week feeding trial.

All birds were housed in an open, ventilated laying house equipped with shaped four-tier stainless steel cages. Each replicate group occupied adjacent cages within the same row. Hens had free access to feed and drinking water throughout the study. During the experimental period, the ambient temperature was maintained at approximately 20 to 22 °C, and the birds were subjected to a daily photoperiod of 16 h of light and 8 h of darkness. The health status and mortality of the hens were monitored at least once daily.

#### 2.3.2. Production Performance and Sample Collection

Laying performance was recorded throughout the 8-week experimental period on a replicate basis. Eggs from each replicate were collected and counted once daily, and the total weight of all eggs laid in each replicate was measured on the same day. Laying rate was expressed as hen day egg production, calculated as the total number of eggs produced in a replicate divided by the number of hens and the number of days, multiplied by 100. Average egg weight was obtained by dividing the total egg weight by the corresponding number of eggs for each replicate and each recording period. Feed offered and residual feed in each replicate were weighed weekly to determine feed intake. Average daily feed intake (ADFI) was calculated as the total feed consumed in a replicate during the week divided by the number of hens and the number of days. Egg mass was calculated as laying rate multiplied by average egg weight for each replicate. The feed conversion ratio was expressed as the ratio of feed intake to egg mass over the same period.

At the end of the 8-week feeding trial, one hen per replicate (8 hens per treatment) was randomly selected for sample collection after 12 h of feed withdrawal, with water remaining available. The live body weight of the selected hens was recorded immediately before slaughter. Blood samples were collected from the wing vein into vacuum tubes, allowed to clot at room temperature, and then centrifuged at 3 000 g for 10 min at 4 °C to obtain serum. The separated serum was transferred into microtubes and stored at −80 °C until analysis. After exsanguination, the abdominal cavity was opened, and the liver, spleen, ovary, and oviduct were carefully removed and weighed. Organ indices were calculated as organ weight divided by live body weight, multiplied by 100 for the liver, spleen, ovary, and oviduct. Segments of approximately 2 cm in length were collected from the middle portion of the duodenum, jejunum, and ileum, gently flushed with PBS to remove luminal contents, and immediately fixed in 4% paraformaldehyde solution for subsequent hematoxylin and eosin (HE) staining and histomorphological evaluation. Cecal contents were collected into sterile tubes, immediately frozen in liquid nitrogen, and then stored at −80 °C for the later determination of short-chain-fatty-acid (SCFA) and microbial compositions. Portions of liver tissue were also sampled, snap frozen in liquid nitrogen, and stored at the same temperature for biochemical and antioxidant analyses.

#### 2.3.3. Egg Quality

Egg quality was assessed in the last week of the trial. For each treatment, 32 eggs were randomly collected on the same day, with 4 eggs taken from each replicate. Eggs were gently cleaned, allowed to equilibrate at about 25 °C for 30 min, and then measured individually. Egg weight was recorded with a precision electronic balance (FA1604N, Shanghai Precision Scientific Instruments Co., Shanghai, China). Egg length and width were measured using a digital vernier caliper, and the egg shape index was calculated as egg width divided by egg length. Eggshell color was determined on the equatorial region using a portable colorimeter (CR-400, Konica Minolta, Tokyo, Japan), and L*, a*, and b* values were read at three different points and averaged for each egg. Albumen height, yolk color score, and Haugh unit were measured using an Egg Multi tester (EMT-5200, Robotmation Co., Tokyo, Japan). Eggshell strength was determined at the equator of the egg using an eggshell force gauge (Model II, Robotmation Co., Tokyo, Japan). After the quality measurements, eggs were carefully broken onto a flat glass plate. Yolks were gently separated from the albumen without damaging the yolk membranes and were immediately weighed. Eggshells were rinsed under running water to remove residual albumen, shell membranes were removed by hand, and the shells were dried in a drying oven at 65 °C for 4 h before weighing to obtain eggshell weight. Eggshell thickness at the blunt end, sharp end, and equatorial region was determined with a dial thickness gauge (P1, Peacock, Ozaki Mfg. Co., Tokyo, Japan), and the mean of the three measurements was taken as the eggshell thickness for each egg. All procedures for egg quality determination were carried out with reference to the NY/T 823-2020 [23] Poultry Performance Measurement Standards, and instruments were calibrated before use to ensure measurement accuracy.

#### 2.3.4. Morphology of Intestinal Mucosa

Segments of the duodenum, jejunum, and ileum fixed in paraformaldehyde were subjected to routine histological processing, including dehydration in a graded ethanol series, clearing in xylene, and embedding in paraffin wax. The embedded tissues were cut into sections of approximately 5 µm, stained with HE, and observed under a digital light microscope equipped with a camera system (BA400Digital, McAudi Industrial Group Co., Ltd., Beijing, China) at 40X magnification. For each intestinal segment, ten well-oriented villus crypt structures were randomly chosen from each sample. Villus height was recorded as the distance from the villus tip to the junction between the villus and the crypt, and crypt depth was measured as the depth of the invagination between two neighboring villi. The ratio of villus height to crypt depth was then calculated to evaluate intestinal morphology.

#### 2.3.5. Serum Biochemistry

Serum biochemical indices, including alkaline phosphatase (ALP), aspartate aminotransferase (AST), urea, alanine aminotransferase (ALT), albumin (ALB), cholesterol (CHO), triglycerides (TG), and total protein (TP), were determined using an automatic serum biochemical analyzer (Hitachi 7020, Hitachi, Tokyo, Japan) with commercial reagent kits purchased from Nanjing Jiancheng Bioengineering Institute (Nanjing, China). Serum concentrations of immunoglobulin A (IgA), immunoglobulin M (IgM), and immunoglobulin G (IgG) were measured using commercially available enzyme-linked immunosorbent assay (ELISA) kits (Nanjing Jiancheng Bioengineering Institute, Nanjing, China).

#### 2.3.6. Antioxidant Capacity

Serum and hepatic antioxidant status were evaluated by determining total antioxidant capacity (T-AOC), catalase activity (CAT), malondialdehyde (MDA) concentration, and total superoxide dismutase (T-SOD) activity. For the liver, approximately 0.10 g of tissue was homogenized in ice-cold physiological saline, and the homogenate was centrifuged to obtain the supernatant. The protein concentration of the liver supernatant was determined using a BCA Protein Assay Kit (Thermo Fisher Scientific, Waltham, MA, USA) according to the manufacturer’s instructions. Serum samples and liver supernatants were then analyzed using commercial kits from Nanjing Jiancheng Bioengineering Institute (Nanjing, China). Hepatic antioxidant indices were normalized to protein concentration and expressed as U/mg protein or nmol/mg protein, whereas serum indices were expressed on a volume basis.

#### 2.3.7. Cecal Short-Chain Fatty Acids

Cecal SCFAs were determined by gas chromatography [24]. Briefly, about 1 g of thawed cecal content was weighed into a centrifuge tube and mixed thoroughly with 4.5 mL of distilled water. The suspension was vortexed, allowed to stand on ice for 30 min, and then centrifuged at 10,000 times g for 10 min at 4 °C. Two milliliters of the supernatant were transferred to a new tube and mixed with 0.5 mL of 25% metaphosphoric acid solution containing an internal standard. After standing for 30 min at 4 °C, the mixture was centrifuged again, and the clear supernatant was passed through a 0.22 μm membrane filter into chromatographic vials. Acetate, propionate, isobutyrate, butyrate, isovalerate, and valerate were analyzed on a gas chromatograph equipped with a flame ionization detector and a capillary fatty-acid column (HP INNOWAX, 30 m × 0.25 mm × 0.25 μm; Agilent Technologies, Santa Clara, CA, USA). The injector and detector temperatures were set at 250 °C. The oven temperature program was as follows: initial temperature 90 °C held for 1 min, then increased to 180 °C at 10 °C per minute, and held for 5 min. Nitrogen was used as the carrier gas. Individual SCFAs were identified by comparing retention times with those of authentic standards and quantified using external standard curves. Because SCFAs were quantified in the clarified supernatant after dilution and acidification, results are presented as mmol/L of the supernatant (extract concentration).

#### 2.3.8. Cecal Microbiota Profiling Analysis

Cecal digesta samples from eight hens in each dietary group (*n* = 8) were used for microbial community analysis. Total bacterial DNA was extracted from approximately 200 mg of cecal content using a commercial DNA extraction kit (Qiagen, Hilden, Germany) following the manufacturer’s instructions. DNA concentration and purity were assessed by 1% agarose gel electrophoresis and a NanoDrop spectrophotometer (Thermo Fisher Scientific, Waltham, MA, USA). The V3–V4 region of the bacterial 16S rRNA gene was amplified using barcoded universal primers 341F (5′-CCTACGGGNGGCWGCAG-3′) and 806R (5′-GGACTACHVGGGTWTCTAAT-3′). Amplicons were purified, quantified, pooled at equimolar concentrations, and sequenced on an Illumina MiSeq platform (2 × 300 bp) by Majorbio Bio-Pharm Technology Co., Ltd. (Shanghai, China). Raw paired-end reads were processed on the Majorbio Cloud Platform following a standardized and fully documented workflow. Briefly, reads were demultiplexed based on unique barcodes, and primer/barcode sequences were trimmed. Quality filtering was performed using fastp (v0.20.0) to remove reads with ambiguous bases, low-quality bases, or insufficient length. High-quality paired-end reads were merged using FLASH (v1.2.11) based on overlap relationships between forward and reverse reads (minimum overlap length ≥ 10 bp; mismatch rate ≤ 0.2). Chimeric sequences were detected and removed using UCHIME (v8.1). After quality control and chimera removal, sequences were clustered into operational taxonomic units (OTUs) at 97% sequence similarity using UPARSE (v7.1), and the most abundant sequence in each OTU was selected as the representative sequence. Taxonomic annotation of representative sequences was conducted using the RDP Classifier (v2.13) against the SILVA 16S rRNA reference database (release 138) with a confidence threshold of 0.70. An OTU table was generated and normalized prior to downstream analyses; alpha and beta diversity analyses were performed on datasets normalized to an even sequencing depth by rarefaction based on the minimum sample sequencing reads after filtering. Alpha diversity indices, including ACE, Chao1, observed OTUs, Shannon index, and coverage, were calculated to describe within-sample richness and diversity. Beta diversity was evaluated using Bray–Curtis distance matrices and visualized by principal coordinate analysis (PCoA). Overall community structural differences among groups were tested using PERMANOVA (adonis) with 999 permutations based on Bray–Curtis distances. Venn diagrams were used to display shared and unique OTUs among groups. Relative abundances of bacterial communities at the phylum and genus levels were summarized and visualized using stacked bar plots. Differentially abundant taxa among dietary groups were identified using the Kruskal–Wallis H test, and when applicable, pairwise comparisons were performed using the Wilcoxon rank-sum test. Spearman’s rank correlation analysis was performed to evaluate associations between microbial genera and serum, liver, and cecal parameters in laying hens. Correlation coefficients (ρ) and corresponding *p* values were calculated, and multiple testing was controlled using the Benjamini–Hochberg false discovery rate (FDR) procedure. Unless otherwise stated, statistical analyses and visualization were conducted in R (v4.2.0).

### 2.4. Statistical Analysis

The data were processed using SAS software, version 9.3 (SAS Institute Inc., Cary, NC, USA). Differences among dietary treatments were assessed by one-way analysis of variance (ANOVA), and linear and quadratic contrasts were used to evaluate dose–response effects. When the overall F test was significant, Duncan multiple range tests were applied for comparison of group means. All results are presented as least squares means with their standard errors. Results with *p* < 0.05 were considered statistically significant, and 0.05 ≤ *p* < 0.10 was interpreted as a tendency.

## 3. Results

### 3.1. Apparent Metabolizable Energy of ZBL

As shown in Table 4, the apparent metabolizable energy of ZBL in laying hen diets was 5.46 MJ/kg, and the nitrogen-corrected apparent metabolizable energy was 5.33 MJ/kg.

### 3.2. Egg Production Performance and Quality

As shown in Table 5, dietary supplementation with 1%, 2%, or 3% ZBL had no significant effects on laying rate, ADFI, average egg weight, feed conversion ratio, or egg mass throughout the experimental period (*p* > 0.05). And in Table 6, albumen height increased linearly with increasing dietary ZBL level (*p* < 0.05), and the Haugh unit showed a linear tendency to increase (*p* = 0.072). Moreover, diets containing 2% and 3% ZBL significantly increased yolk color score compared with the control (*p* < 0.05).

### 3.3. Serum Biochemical Parameters of Laying Hens

**Figure 1:**
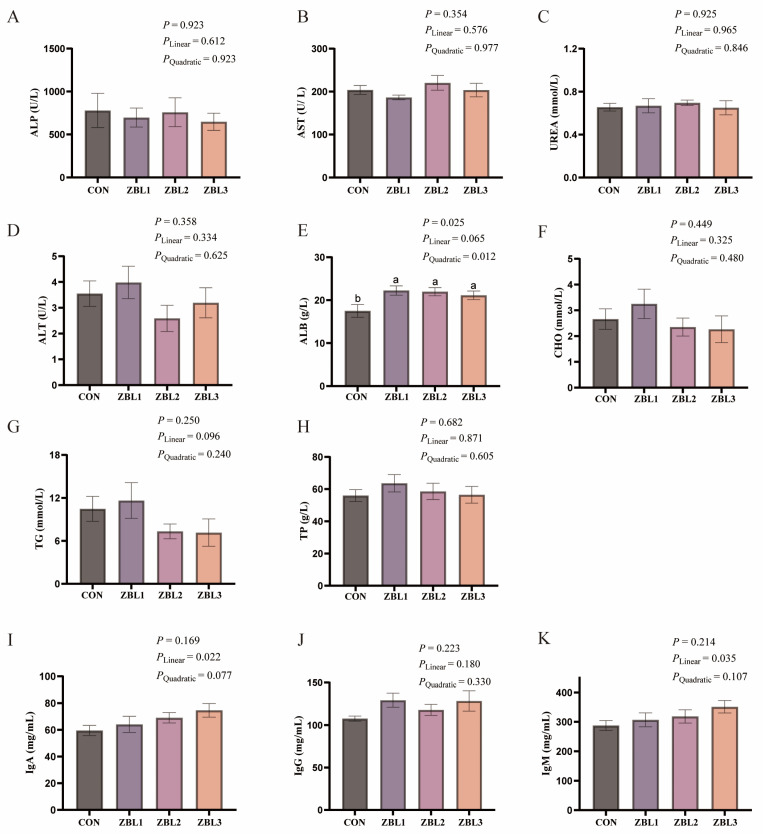
Effects of dietary ZBL supplementation on serum biochemical parameters in laying hens. (**A**) Alkaline phosphatase (ALP), (**B**) aspartate aminotransferase (AST), (**C**) urea, (**D**) alanine aminotransferase (ALT), (**E**) albumin (ALB), (**F**) cholesterol (CHO), (**G**) triglyceride (TG), (**H**) total protein (TP), (**I**) immunoglobulin A (IgA), (**J**) immunoglobulin G (IgG), and (**K**) immunoglobulin M (IgM) in the serum of laying hens fed diets supplemented with 0 (CON), 1% (ZBL1), 2% (ZBL2), or 3% (ZBL3) ZBL. Different letters indicate significant differences among groups (*p* < 0.05). Linear and quadratic effects are also indicated. Data are presented as mean ± SEM (*n* = 8).

As shown in Figure 1, dietary supplementation with ZBL significantly increased serum ALB concentration compared with the control (*p* < 0.05), and ALB showed a linear tendency to increase with rising dietary ZBL level (*p* = 0.065). In addition, ZBL supplementation significantly increased serum IgA and IgM concentrations in a linear manner (*p* < 0.05).

### 3.4. Organ Indices of Laying Hens

**Figure 2:**
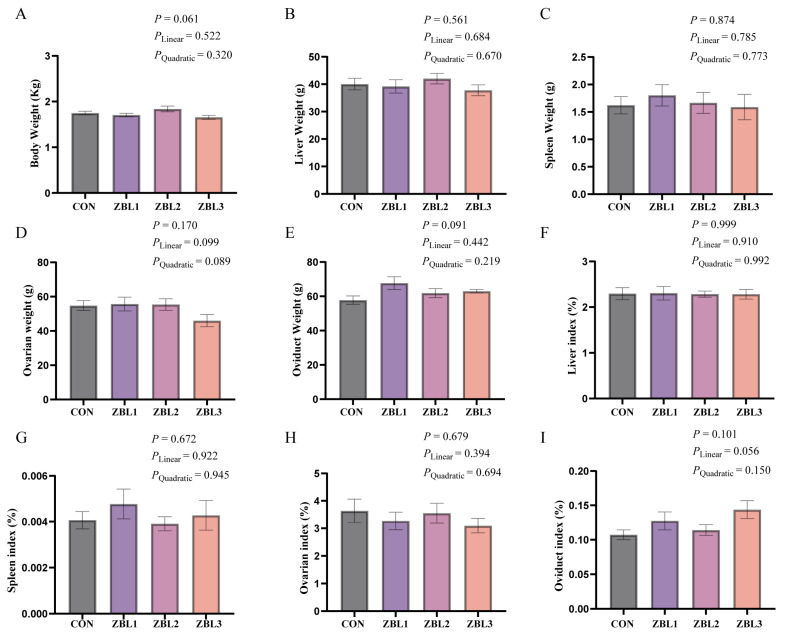
Effects of dietary ZBL supplementation on organ indices in laying hens. (**A**) Body weight, (**B**) liver weight, (**C**) spleen weight, (**D**) ovary weight, (**E**) oviduct weight, (**F**) liver index, (**G**) spleen index, (**H**) ovary index, and (**I**) oviduct index of laying hens fed diets with 0 (CON), 1% (ZBL1), 2% (ZBL2), or 3% (ZBL3) ZBL. Organ index = organ weight (g)/body weight (g). Linear and quadratic effects are shown for reference. Data are presented as mean ± SEM (*n* = 8).

As shown in Figure 2, dietary supplementation with ZBL tended to increase body weight (*p* = 0.061) and oviduct weight (*p* = 0.091) of laying hens. Moreover, the oviduct index showed a tendency to increase linearly with increasing dietary ZBL level (*p* = 0.056).

### 3.5. Serum Antioxidant Capacity of Laying Hens

**Figure 3:**
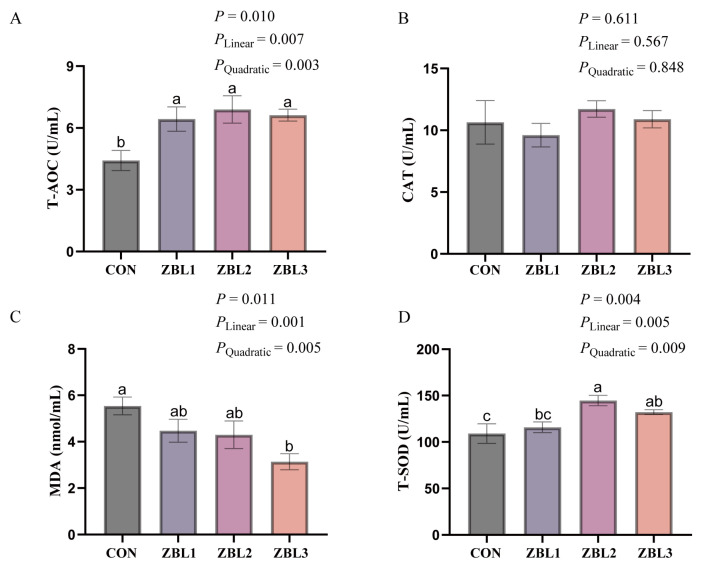
Effects of dietary ZBL supplementation on serum antioxidant parameters in laying hens. (**A**) Total antioxidant capacity (T-AOC), (**B**) catalase activity (CAT), (**C**) malondialdehyde content (MDA), (**D**) total superoxide dismutase activity (T-SOD) in the serum of laying hens fed diets with 0 (CON), 1% (ZBL1), 2% (ZBL2), or 3% (ZBL3) ZBL. Different letters indicate significant differences among groups (*p* < 0.05). Linear and quadratic effects are also presented. Data are presented as mean ± SEM (*n* = 8).

As shown in Figure 3, dietary supplementation with ZBL significantly increased serum T-AOC (*p* < 0.05) and T-SOD (*p* < 0.05) activities. Both indices showed significant linear and quadratic responses to increasing dietary ZBL level (*p* < 0.05). Compared with the control, diets containing 1%, 2%, and 3% ZBL all elevated T-AOC, whereas T-SOD activity was highest in hens fed 2% ZBL and decreased slightly at 3% but remained higher than in the control. In contrast, serum MDA concentration was significantly reduced by ZBL supplementation (*p* < 0.05) and exhibited both linear and quadratic decreases (*p* < 0.05), with the lowest value observed in the 3% ZBL group.

### 3.6. Hepatic Antioxidant Status of Laying Hens

**Figure 4:**
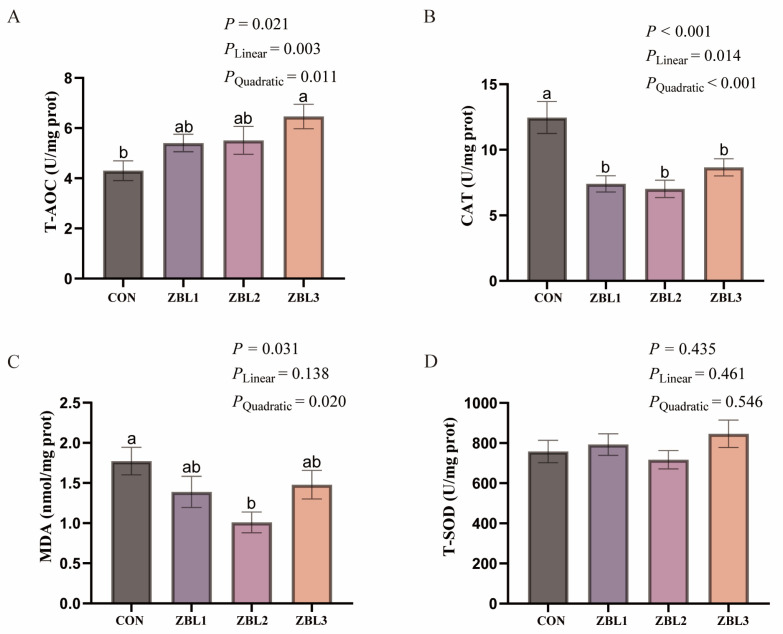
Effects of dietary ZBL supplementation on liver antioxidant capacity in laying hens. (**A**) Total antioxidant capacity (T-AOC), (**B**) catalase activity (CAT), (**C**) malondialdehyde content (MDA), and (**D**) total superoxide dismutase activity (T-SOD) in the serum of laying hens fed diets with 0 (CON), 1% (ZBL1), 2% (ZBL2), or 3% (ZBL3) ZBL. Different letters indicate significant differences among groups (*p* < 0.05). Linear and quadratic effects are also indicated. Data are presented as mean ± SEM (*n* = 8).

As shown in Figure 4, hens fed 3% ZBL had significantly higher hepatic T-AOC than the control group (*p* < 0.05) and showed significant linear and quadratic responses to increasing dietary ZBL level (*p* < 0.05). Hepatic CAT activity was highest in the control hens and was significantly lower in all ZBL-supplemented groups (*p* < 0.05), with both linear and quadratic effects (*p* < 0.05). Hepatic MDA concentration was also decreased by ZBL supplementation (*p* < 0.05) and exhibited a significant quadratic pattern (*p* < 0.05), with the lowest value observed in the 2% ZBL group.

### 3.7. Cecal Short-Chain Fatty Acids

**Figure 5:**
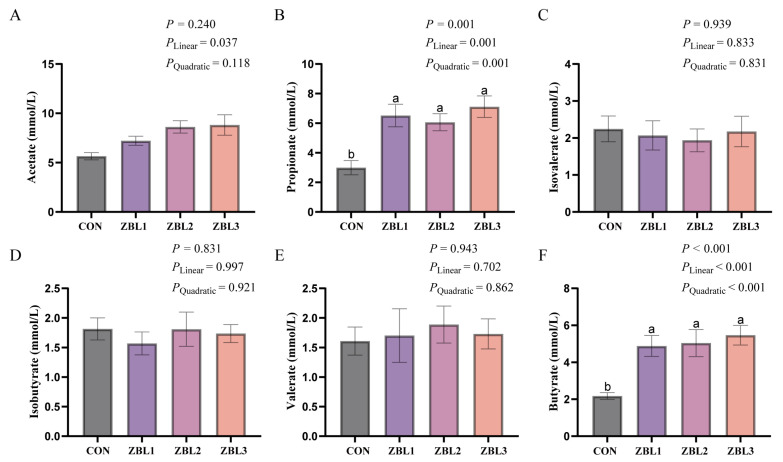
Effects of dietary ZBL supplementation on cecal SCFAs concentrations in laying hens. (**A**) Acetate, (**B**) propionate, (**C**) isobutyrate, (**D**) isobutyrate, (**E**) valerate, and (**F**) butyrate in the cecal contents of hens fed diets containing 0 (CON), 1% (ZBL1), 2% (ZBL2), or 3% (ZBL3) ZBL. Different letters above bars indicate significant differences among groups (*p* < 0.05). Linear and quadratic effects are also presented. Data are presented as mean ± SEM (*n* = 8).

Dietary ZBL supplementation markedly altered several cecal SCFAs (Figure 5). Acetate concentration showed a significant linear increase with rising dietary ZBL level (*p* < 0.05). Propionate concentration was significantly elevated by ZBL supplementation (*p* < 0.05), with both linear and quadratic effects (*p* < 0.05); all ZBL-treated groups showed higher propionate levels than the control. Similarly, butyrate concentration was significantly increased by dietary ZBL (*p* < 0.05), exhibiting clear linear and quadratic dose response patterns (*p* < 0.05), and all three supplementation levels resulted in higher butyrate than the control.

### 3.8. Intestinal Morphology

**Figure 6:**
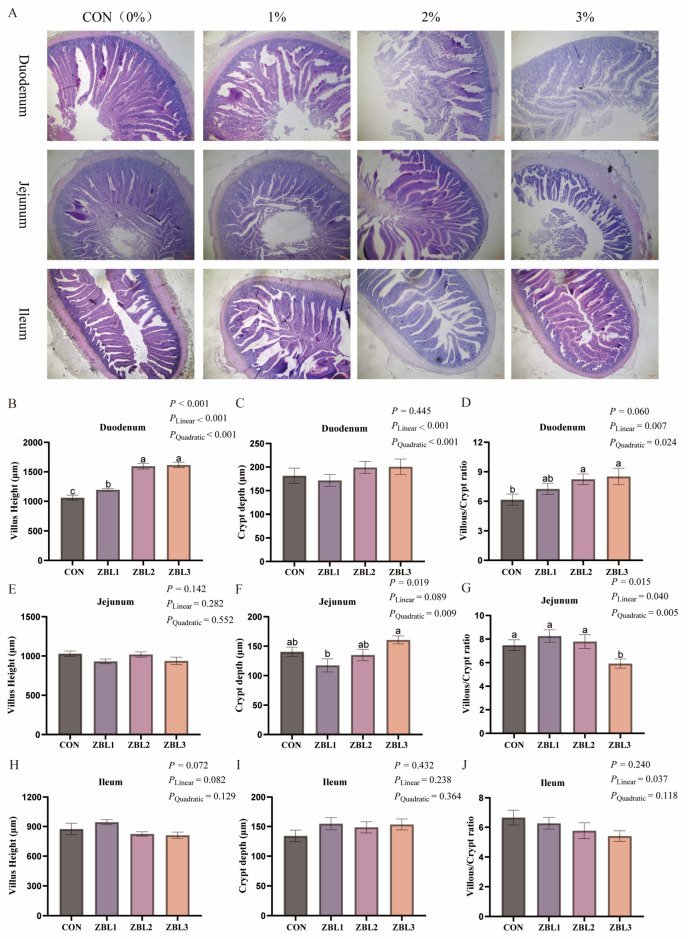
Effects of dietary ZBL supplementation on intestinal morphology in laying hens. (**A**) Representative histological images of the duodenum, jejunum, and ileum from hens fed diets with 0 (CON), 1% (ZBL1), 2% (ZBL2), or 3% (ZBL3) ZBL (H&E staining, scale bar = 200 μm). Images are representative; no post-acquisition enhancement was applied. (**B**–**J**) Morphometric analysis of intestinal segments: villus height (**B**,**E**,**H**), crypt depth (**C**,**F**,**I**), and villus-height-to-crypt-depth ratio (**D**,**G**,**J**) in the duodenum, jejunum, and ileum, respectively. Different letters above bars indicate significant differences (*p* < 0.05). *p*-values for linear and quadratic trends are also shown. Data are presented as mean ± SEM (*n* = 8).

As shown in Figure 6, dietary ZBL supplementation markedly affected small intestinal morphology. In the duodenum, villus height was significantly increased by ZBL (*p* < 0.05) and showed both linear and quadratic dose response patterns (*p* < 0.05), with all supplemented groups having higher villi than the control and the greatest values at 2% and 3% ZBL. The V/C ratio in the duodenum tended to increase with rising ZBL level (*p* = 0.060), and was higher in the 2% and 3% groups than in the control. In the jejunum, ZBL significantly influenced crypt depth (*p* < 0.05), with hens fed 1% ZBL showing lower crypts than those fed 3%. The jejunal V/C ratio was also significantly decreased in the 3% ZBL group, displaying both linear and quadratic responses to increasing ZBL inclusion (*p* < 0.05). In the ileum, villus height showed a tendency to increase with dietary ZBL (*p* = 0.072), whereas crypt depth was not altered. The ileal V/C ratio exhibited a linear decrease with ZBL level (*p* < 0.05).

### 3.9. Cecum Microbiota Diversity

**Figure 7:**
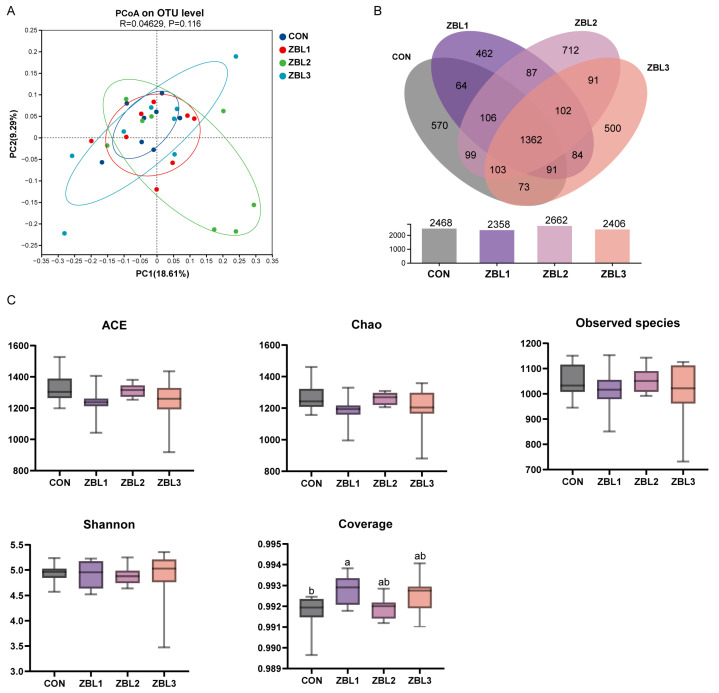
Effects of ZBL treatment on gut microbiota diversity and richness in laying hens. (**A**) Principal coordinate analysis (PCoA) based on OTU level showing beta diversity among control (CON) and ZBL-treated groups (ZBL1, ZBL2, ZBL3). (**B**) Venn diagram and bar plot indicating shared and unique OTUs among groups. (**C**) Alpha diversity indices, including ACE, Chao1, observed species, Shannon, and coverage. Different letters above bars indicate significant differences (*p* < 0.05). *n* = 8.

In the PCoA plot based on OTU level distances (Figure 7A), samples from the control group and from hens fed 1%, 2%, and 3% ZBL showed substantial overlap, and no clear separation among dietary treatments was observed. As shown in Figure 7B, the Venn diagram indicated that 2468, 2358, 2662, and 2406 OTUs were detected in the CON, ZBL1, ZBL2, and ZBL3 groups, respectively, with 1362 OTUs shared by all four groups. Alpha diversity indices, including ACE, Chao, observed species, and Shannon (Figure 7C), were generally similar among the four treatments. Sequencing coverage was significantly higher in the ZBL1 group than in the CON group (*p* < 0.05).

### 3.10. Cecum Microbiota Composition

**Figure 8:**
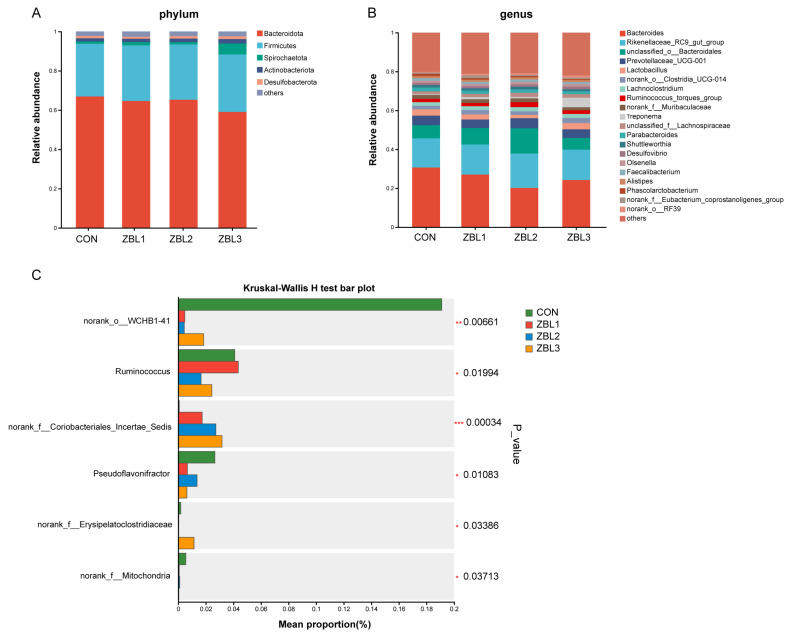
Effects of ZBL treatment on gut microbiota composition in laying hens. (**A**) Relative abundance of gut microbiota at the phylum level in control (CON) and ZBL-treated groups (ZBL1, ZBL2, and ZBL3). (**B**) Relative abundance at the genus level among groups. (**C**) Kruskal–Wallis H test showing significantly different taxa among groups. Bars represent mean relative abundance (%), with *p*-values labeled. * *p* < 0.05, ** *p* < 0.01, *** *p* < 0.001, *p * <  0.05 is considered significant (red asterisks). *n* = 8.

At the phylum level (Figure 8A), *Bacteroidota* and *Firmicutes* dominated the cecal microbiota in all groups, and the overall phylum level composition was similar among CON, ZBL1, ZBL2, and ZBL3. At the genus level (Figure 8B), *Bacteroides* and other common genera were predominant, and no large shifts in the main genera were observed with ZBL inclusion. Kruskal–Wallis analysis (Figure 8C) showed that several low-abundance taxa differed among treatments (*p* < 0.05). The relative abundance of *norank_o__WCHB1-41* was highest in the CON group and was markedly reduced in all ZBL groups. In contrast, the genera *Ruminococcus*, *Pseudoflavonifractor*, and members of *norank_f__Coriobacteriales_Incertaesedis*, *norank_f__Erysipelatoclostridiaceae*, and *norank_f__Mitochondria* were increased in hens receiving ZBL diets compared with the control.

### 3.11. Correlation Analysis

**Figure 9:**
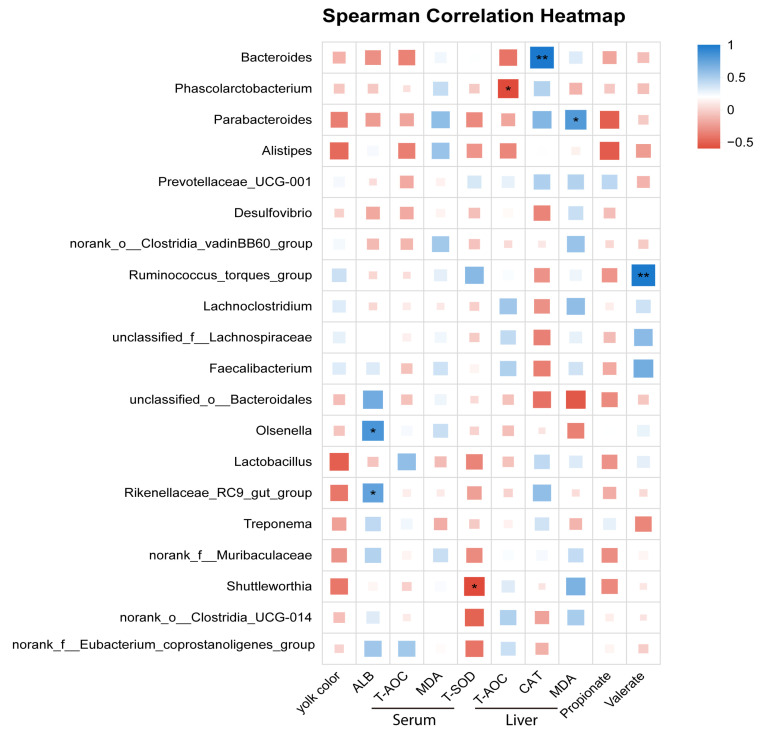
Spearman correlation heatmap between microbial genera and serum, liver, and cecal parameters in laying hens. The heatmap shows the Spearman correlation coefficients between the relative abundance of selected bacterial genera and physiological or biochemical parameters, including yolk color, serum indices (ALB, T-AOC, MDA, and T-SOD), liver antioxidant parameters (T-AOC, CAT, and MDA), and cecal SCFAs (propionate, valerate). Red and blue indicate negative and positive correlations, respectively. The intensity of the color reflects the strength of the correlation. * indicates *p* < 0.05 and ** indicates *p*  <  0.01. The square size is proportional to the absolute Spearman’s correlation coefficient (|ρ|), with larger squares indicating stronger correlations. *n* = 8.

As shown in Figure 9, serum ALB was positively associated with the genera *Rikenellaceae_RC9_gut_group* and *Olsenella* (*p* < 0.05). Serum T-SOD showed a significant negative correlation with *Shuttleworthia* (*p* < 0.05). In the liver, T-AOC was negatively correlated with *Phascolarctobacterium* (*p* < 0.05), whereas hepatic CAT activity was positively correlated with *Bacteroides* (*p* < 0.05). Hepatic MDA concentration was positively associated with *Parabacteroides* (*p* < 0.05). In addition, the cecal valerate concentration exhibited a significant positive correlation with *Ruminococcus_torques_group* (*p* < 0.05).

## 4. Discussion

With the increasing competition between humans and livestock for cereal grains and the rising cost of conventional feed ingredients, there is growing interest in exploiting locally available plant byproducts and non-grain resources as alternative ingredients in poultry diets [25,26]. Accurate estimation of the energy value of unconventional feed ingredients is a prerequisite for their rational use in layer diets and for avoiding unintended dilution of dietary energy. In this context, the present study shows that the AME and AMEn of ZBL for laying hens are 5.46 MJ/kg and 5.33 MJ/kg, respectively, which clearly positions ZBL below cereals in energy density but within the range reported for other leafy materials such as mulberry leaf meal in poultry [27]. This energetic profile means that ZBL is more appropriately regarded as a fibrous, bioactive component than as a major contributor of metabolizable energy. The relatively small gap between AME and AMEn also suggests that nitrogen retention from ZBL is limited, reinforcing the view that its nutritional value lies less in protein or energy supply and more in its non-nutritive constituents. Against this background, the absence of any detrimental effect of 1–3% ZBL on laying rate, feed intake, egg weight, or feed conversion ratio becomes informative. Rather than being a trivial negative result, it indicates that when ZBL is used to replace a low-energy ingredient, such as wheat bran, and the diet is properly balanced, its inclusion does not impose an energetic or palatability penalty on hens. This is consistent with work on other leaf-based ingredients and phytogenic additives in layers, where moderate inclusion levels rarely impair production provided that energy and amino acid densities are maintained [28]. Recent work in broilers showed that 1–2% ZBL improved antioxidant status and intestinal barrier function without compromising growth, whereas higher levels could begin to depress performance [7]. Our data extend these findings to laying hens, suggesting that 1–3% ZBL is within a physiologically acceptable range when used as a partial replacer of low-energy byproducts.

More importantly, the egg quality data suggest that ZBL acted on egg formation rather than simply diluting or replacing nutrients in the diet. Although eggshell color is largely genetically determined, its pigmentation intensity can vary with the hen’s physiological status, along with environmental and management factors [29]. Therefore, L*, a*, and b* values were recorded as an objective shell-quality trait. The linear increase in albumen height and the tendency for higher Haugh units occurred without any change in egg weight, which points to better preservation of albumen structure rather than a trivial effect of smaller eggs. A reasonable explanation is that the flavonoids and other polyphenols in ZBL help to limit oxidative damage to albumen proteins during follicular development and passage through the oviduct, a mechanism that has also been proposed for other polyphenol-rich or phytogenic additives that improve albumen quality in laying hens [30]. The stronger yolk color at 2–3% ZBL inclusion is consistent with the presence of pigmented flavonoids and related compounds and aligns with findings that flavonoid-rich plant byproducts can enhance yolk color, modulate yolk lipid profiles, and improve flavor without compromising laying rate [31]. Since yolk color is a key trait for consumer acceptance and price differentiation of table eggs, the combination of unchanged egg production with improved albumen quality and yolk pigmentation indicates that ZBL has the potential to turn a low-value orchard byproduct into a functional feed ingredient that adds both nutritional and commercial value to eggs.

The serum biochemical response observed in the current study suggests a modest improvement in systemic nutritional and immune status rather than random variation. ALB, synthesized by the liver, is a key marker of protein nutrition and hepatic function. Its dose-dependent increase with ZBL inclusion, without changes in feed intake or egg output, implies more efficient nutrient utilization. Similar ALB elevations have been reported in hens supplemented with traditional Chinese herbal residues, often alongside improved hepatic antioxidant capacity [32]. Given that ZBL contains flavonoids such as quercetin and apigenin, which support hepatic antioxidant defense, the higher ALB likely reflects enhanced hepatic metabolic resilience rather than increased protein intake [33]. The linear increases in serum IgA and IgM further support an immunomodulatory role of ZBL. IgA and IgM are key components of mucosal and systemic humoral immunity in chickens, and higher circulating levels are generally associated with enhanced immune readiness under both normal and stress conditions [34]. Studies with quercetin, tea polyphenols, and traditional Chinese herbal mixtures have consistently shown that dietary polyphenol-rich preparations can raise serum IgA, IgM, and IgG in layers, while simultaneously lowering pro-inflammatory cytokines such as TNF-α and IL-1β [35]. The present data fit well into this pattern and suggest that the flavonoid and polyphenol fraction of ZBL does not simply act as an antioxidant but also fine-tunes immune function in clinically healthy hens.

The organ index data, although showing trends rather than marked significance, are consistent with this interpretation. The numerical increases in body weight and oviduct weight, together with the tendency for a higher oviduct index, suggest that ZBL may help maintain reproductive tract function rather than simply increase carcass mass. Similar effects have been observed with phytogenic or polyphenol-based additives, which can enhance ovarian and oviductal development, often in association with improved egg quality and, in some cases, elevated estradiol levels in aging hens [36]. These effects have been linked to a combination of reduced oxidative stress in reproductive tissues and mild phytoestrogen-like activity of certain plant flavonoids [37]. ZBL contains multiple flavonoid structures with known estrogenic or hormone-modulating potential [38]. In the present study, ZBL also enhanced antioxidant capacity in serum and liver. It is therefore plausible that the slight enlargement of the oviduct reflects a more favorable oxidative and endocrine environment for the reproductive tract, even though the magnitude of the effect was not sufficient to translate into a detectable change in laying rate within the eight-week trial.

The changes in serum and hepatic antioxidant indices collectively indicate that ZBL supplementation created a more favorable systemic redox environment, although the response was not strictly dose-proportional. In serum, T-AOC and T-SOD activities were consistently elevated while MDA concentrations decreased across all ZBL treatments, with the strongest responses generally at 2–3% inclusion. This antioxidant profile, characterized by higher total antioxidant capacity and SOD activity along with lower lipid peroxidation, is typical of birds supplemented with polyphenol-rich or herbal additives and is usually interpreted as a genuine enhancement of endogenous antioxidant defense rather than a random analytical fluctuation [39]. Given that ZBL is rich in quercetin, apigenin glycosides, and other flavonoid structures with proven free radical scavenging and metal chelating activities, it is reasonable to infer that the improved antioxidant status reflects both direct radical quenching activity and an upregulation of enzymatic antioxidant systems [40]. Indeed, recent work in broilers confirmed that dietary ZBL enhances antioxidant capacity through Nrf2/HO-1 pathway activation, elevating T-AOC, T-SOD, and CAT activities in both serum and liver [7]. In the liver, the response was more nuanced but still consistent with reduced oxidative burden. Hepatic T-AOC increased significantly at 3% ZBL, while MDA showed a quadratic decline with the lowest values around 2%, indicating enhanced protection against lipid peroxidation in this metabolically active organ. This interpretation aligns with findings from both *Zanthoxylum* extracts and other flavonoid sources, where T-AOC and SOD are upregulated while MDA is reduced and CAT activity tends to normalize or decline under reduced oxidative stress [41,42].

Dietary ZBL supplementation also exerted distinct effects on the distal gut environment. The consistent increases in cecal acetate, propionate, and butyrate concentrations suggest that fermentative activity was enhanced, even though overall α- and β-diversity of the cecal microbiota remained largely unchanged. In poultry, these SCFAs serve not only as important energy sources for colonocytes but also as key signaling molecules regulating epithelial proliferation, barrier integrity, and immune responses [43]. Butyrate, in particular, is known to promote tight-junction protein expression, increase mucus secretion, and suppress proinflammatory signaling in the avian gut [44]. The higher SCFA concentrations observed in ZBL-fed hens, therefore, align with the observed improvements in intestinal morphology and immune indices, suggesting that ZBL fostered a more metabolically active and functionally supportive cecal milieu. The histomorphological changes in the small intestine support this interpretation. Duodenal villus height was significantly increased across all ZBL levels, with a trend toward a higher V/C ratio at moderate inclusion levels, indicative of an enlarged absorptive surface and lower epithelial turnover [45]. Similar patterns have been reported in layers receiving polyphenol- or flavonoid-rich additives, such as chlorogenic acid or mixed herbal powders, which enhance villus structure and promote gut integrity [46,47]. The dose-dependent response observed in jejunal crypt depth and V/C ratio further indicates that 3% ZBL may approach the upper limit of a beneficial range, consistent with the concept that moderate levels of fermentable fiber and polyphenols optimize mucosal efficiency, whereas excessive levels may slightly disturb epithelial homeostasis [48]. The ileal changes, although less pronounced, were consistent with a mild trophic effect extending along the distal small intestine and corresponded with the higher cecal SCFAs production. These adaptations occurred without notable changes in overall microbiota diversity. Principal coordinate analysis and α-diversity indices showed no clear dietary clustering, indicating that ZBL did not markedly alter cecal community composition. Similar observations have been reported in hens supplemented with polyphenol-rich additives or xylo-oligosaccharides, where fermentative activity increased despite stable microbial diversity [49]. Consistent with these functional outcomes, the analysis of cecal microbiota composition revealed that *Bacteroidota* and *Firmicutes* remained the dominant phyla across all groups, indicating that ZBL supplementation did not disrupt the core microbial structure. However, several low-abundance taxa responded significantly to dietary ZBL, suggesting selective modulation of microbial metabolism rather than large-scale compositional shifts. The increased relative abundance of *Ruminococcus* and *Pseudoflavonifractor*, along with members of *Coriobacteriales*_*Incertaesedis* and *Erysipelatoclostridiaceae*, is noteworthy since these taxa are involved in the fermentation of plant polysaccharides and the production of SCFAs, particularly butyrate, which supports epithelial integrity and immune regulation [50]. The decline in *norank_o__WCHB1-41* abundance in all ZBL groups may reflect a shift away from proteolytic fermentation toward a more saccharolytic and beneficial microbial metabolism. Such targeted microbial adjustments are consistent with the observed increase in cecal SCFAs and improved intestinal morphology, further supporting the idea that ZBL acts as a mild but effective modulator of gut fermentation processes. We further linked the gut microbiota with host health-related indices, and found a clear positive correlation between cecal valerate and *Ruminococcus_torques_group*. Valerate has been shown to strengthen epithelial barrier function by increasing TEER and lowering paracellular permeability in Caco-2 monolayers [51]. More broadly, SCFAs can support barrier integrity and modulate inflammation through tight junction regulation [52]. In our study, serum and hepatic antioxidant indices also correlated with some genera, suggesting microbiota shifts and redox status moved together under the ZBL diets. This fits with poultry evidence that ZBL powder can raise CAT, T-AOC, and T-SOD and activate Nrf2/HO-1 signaling [7].

In addition, we acknowledge that the phytochemical composition of ZBL may vary with cultivar, harvest season, maturity, growing region, and post-harvest processing conditions such as drying and storage. Such variation may partly explain differences in responses when ZBL is produced from different sources. Therefore, although our data support ZBL as a functional feed ingredient and indicate a practical inclusion range of 2–3% under the present conditions, further studies using multiple batches from different harvests and processing procedures would be helpful to confirm the robustness of this recommendation. Reporting key marker compounds and basic processing information would also facilitate comparison across studies and improve practical application.

## 5. Conclusions

This study found that ZBL can be positioned as a low-energy but bioactive feed ingredient for laying hens rather than a primary energy source. Integrating production performance, egg quality, antioxidant indices, SCFAs, and intestinal morphology, 2–3% ZBL was identified as the recommended inclusion range in balanced layer diets. Within this range, ZBL maintained laying performance while improving egg quality, enhancing antioxidant status, and benefiting gut health by improving intestinal morphology, enriching SCFA-producing microbes, and increasing cecal SCFAs concentrations. These findings suggest that ZBL offers a feasible way to upgrade an underutilized orchard byproduct into a functional ingredient for the egg industry.

## Figures and Tables

**Table 1:** Nutrient composition of ZBL (on a dry-matter basis).

Items	DM/%	CP/%	EE/%	CF/%	Ash/%	Ca/%	TP/%	Total Flavone/(g/kg)	Total Polyphenol/(g/kg)	GE/(MJ/kg)
Content	90.20	14.02	2.41	13.30	12.30	3.34	0.14	53.31	43.37	16.57

DM—dry matter; CP—crude protein; EE—ether extract; CF—crude fiber; Ca—Calcium; TP—total phosphorus; GE—gross energy.

**Table 2:** Composition and nutrient levels of diets in experiment 1 (air-dry basis, %).

Items	Basal Diet
Ingredients	
Corn	65.20
Soybean meal	24.00
*Zanthoxylum bungeanum* leaves	—
NaCl	0.30
Limestone	8.50
Calcium phosphate	1.50
DL-Methionine	0.17
Mineral premix ^1^	0.30
Vitamin premix ^2^	0.03
**Total**	100.00
**Nutrient levels ^3^**	
Gross energy, MJ/kg	14.71
Crude protein	16.64
Calcium	3.71
Total phosphorus	0.60
Lysine	0.73
Methionine	0.39

^1^ The mineral premix provided the following per kg of diets: Cu (CuSO_4_·5H_2_O) 8 mg, Fe (FeSO_4_·H_2_O) 60 mg, Mn (MnSO_4_·H_2_O) 60 mg, Zn (ZnSO_4_·H_2_O) 80 mg, I (KI) 0.35 mg, Se (Na_2_SeO_3_) 0.30 mg. ^2^ The vitamin premix provided the following per kg of diets: VA 8000 IU, VD_3_ 1600 IU, VE 5 IU, VB_1_ 0.8 mg, VB_2_ 2.5 mg, D-calcium pantothenate 2.2 mg, VB_3_ 3.0 mg, VB_6_ 0.004 mg, VK 0.5 mg, biotin 0.10 mg, niacin 20.0 mg. ^3^ Lys and Met were calculated values. Other nutrient values were measured. Nutritional values were determined based on standards from Tables of Feed Composition and Nutritional Values in China (2024, 35th edition) [22], GE (ISO 9831:1998) [17], CP (GB/T 6432-2018) [11], Ca (GB/T 6436-2018) [15], and TP (GB/T 6437-2018) [16].

**Table 3:** Composition and nutrient levels of diets in experiment 2 (air-dry basis, %).

Items	Supplemental Levels of ZBL/%			
	Control (0%)	1%	2%	3%
Ingredients				
Corn	60.70	60.20	59.70	59.20
Soybean meal	22.00	21.50	21.00	20.50
Wheat bran	3.00	3.00	3.00	3.00
Soybean oil	3.00	3.00	3.00	3.00
Limestone	8.97	8.97	8.97	8.97
*Zanthoxylum bungeanum* leaves	—	1.00	2.00	3.00
Dicalcium phosphate	1.40	1.40	1.40	1.40
L-Lysine·HCl	0.13	0.13	0.13	0.13
DL-Methionine	0.16	0.16	0.16	0.16
L-Threonine	0.02	0.02	0.02	0.02
Sodium chloride	0.25	0.25	0.25	0.25
Sodium bicarbonate	0.10	0.10	0.10	0.10
Choline chloride	0.10	0.10	0.10	0.10
Mineral premix ^1^	0.15	0.15	0.15	0.15
Vitamin premix ^2^	0.02	0.02	0.02	0.02
**Total**	100.00	100.00	100.00	100.00
**Nutrient levels ^3^**				
Metabolizable energy, MJ/kg	11.60	11.53	11.47	11.41
Crude protein	16.77	16.65	16.53	16.41
Calcium	3.86	3.89	3.92	3.96
Total phosphorus	0.58	0.58	0.58	0.57
Available phosphorus	0.32	0.32	0.32	0.32
Digestible lysine	0.79	0.78	0.77	0.77
Digestible methionine	0.37	0.37	0.36	0.36
Digestible threonine	0.50	0.50	0.49	0.49
Digestible tryptophan	0.15	0.15	0.14	0.14

^1^ Mineral premix provided the following per kg of diets: Cu (CuSO_4_·5H_2_O) 8 mg, Fe (FeSO_4_·H_2_O) 60 mg, Mn (MnSO_4_·H_2_O) 60 mg, Zn (ZnSO_4_·H_2_O) 80 mg, I (KI) 0.35 mg, Se (Na_2_SeO_3_) 0.30 mg. ^2^ Vitamin premix provided the following per kg of diets: VA 8000 IU, VD_3_ 1600 IU, VE 5 IU, VB_1_ 0.8 mg, VB_2_ 2.5 mg, D-calcium pantothenate 2.2 mg, VB_3_ 3.0 mg, VB_6_ 0.004 mg, VK_3_ 0.5 mg, biotin 0.10 mg, nicotinic acid 20.0 mg. ^3^ Crude protein (CP) and available phosphorus (AP) were measured values. The other values were calculated based on the “Tables of Feed Composition” and Nutritive Values in China (35th edition, 2024) [22].

**Table 4:** Apparent metabolizable energy and nitrogen-corrected apparent metabolizable energy of ZBL (air-dry basis).

Item	AME, MJ/kg	AMEn, MJ/kg
ZBL	5.46	5.33

AME—apparent metabolizable energy; AMEn—nitrogen-corrected apparent metabolizable energy.

**Table 5:** Effects of different dietary inclusion levels of ZBL on laying hens’ egg production performance.

Items	ZBL				SEM	*p*-Value		
	0 (CON)	1%	2%	3%		ANOVA	Linear	Quadratic
Weeks 1–4								
Laying rate, %	91.54	90.75	90.88	91.87	0.596	0.506	0.672	0.305
ADFI, g/d/hen	109.39	112.99	113.30	113.59	2.388	0.571	0.224	0.378
Average egg weight, g	59.39	59.53	59.93	61.33	1.107	0.594	0.205	0.385
Feed conversion ratio	2.12	2.13	2.15	2.07	0.050	0.676	0.503	0.523
Egg mass, g/hen/day	54.38	54.03	54.49	56.37	1.175	0.505	0.224	0.306
Weeks 5–8								
Laying rate, %	89.89	89.56	89.45	89.86	0.962	0.984	0.960	0.923
ADFI, g/d/hen	112.12	115.95	114.47	113.55	1.697	0.453	0.717	0.169
Average egg weight, g	58.26	58.38	59.17	59.67	1.414	0.878	0.417	0.716
Feed conversion ratio	2.26	2.19	2.21	2.11	0.061	0.356	0.105	0.261
Egg mass, g/hen/day	52.42	52.33	52.29	53.68	1.596	0.927	0.529	0.793
Weeks 1–8								
Laying rate, %	90.17	90.15	90.17	90.87	0.603	0.773	0.860	0.566
ADFI, g/d/hen	110.75	114.47	113.89	113.57	1.927	0.539	0.363	0.384
Average egg weight, g	58.82	58.95	59.55	60.50	1.178	0.740	0.279	0.528
Feed conversion ratio	2.19	2.16	2.18	2.09	0.054	0.529	0.231	0.409
Egg mass, g/hen/day	53.39	53.16	53.70	55.01	1.242	0.723	0.325	0.510

ADFI—average daily feed intake; CON—control diet; SEM—standard error of the mean. *n* = 8.

**Table 6:** Effects of different dietary inclusion levels of ZBL on egg quality.

Items	ZBL				SEM	*p*-Value		
	0 (CON)	1%	2%	3%		ANOVA	Linear	Quadratic
Eggshell color								
L*	78.39	77.10	77.66	79.27	1.150	0.579	0.536	0.371
a*	5.69	5.17	5.07	5.55	0.321	0.469	0.725	0.277
b*	14.57	14.53	14.33	15.55	0.561	0.429	0.280	0.300
Egg shape index	1.50	1.42	1.47	1.41	0.052	0.579	0.337	0.634
Eggshell strength, kg/cm^2^	4.34	4.64	4.35	4.82	0.212	0.325	0.236	0.465
Egg weight, g	59.64	61.08	61.73	60.59	0.951	0.477	0.419	0.290
Albumen height, mm	7.11	7.50	7.47	7.90	0.220	0.118	0.022	0.075
Yolk color	4.71 ^b^	5.17 ^b^	6.01 ^a^	5.94 ^a^	0.191	<0.001	<0.001	<0.001
Haugh unit	84.16	86.08	84.88	88.48	1.414	0.171	0.072	0.172
Yolk weight, g	16.28	16.53	16.59	16.61	0.177	0.537	0.183	0.338
Eggshell weight, g	5.95	6.29	6.19	6.18	0.112	0.204	0.280	0.168
Eggshell thickness, mm	0.35	0.36	0.35	0.35	0.006	0.650	0.930	0.807

CON—control diet; SEM—standard error of the mean. *n* = 8. ^a,b^ Mean values within a row with different superscript letters are significantly difference (*p* < 0.05).

## Data Availability

None of the data were deposited in an official repository. Data can be made available from the authors upon request.

## Abbreviations

The following abbreviations are used in this manuscript:

ZBL	*Zanthoxylum bungeanum* leaves
CP	Crude protein
Ca	Calcium
P	Phosphorus
AME	Apparent metabolizable energy
AMEn	Nitrogen-corrected apparent metabolizable energy
ADFI	Average daily feed intake
HE	Haematoxylin and eosin
SCFAs	Short-chain fatty acids
ALP	Alkaline phosphatase
AST	Aspartate aminotransferase
ALT	Alanine aminotransferase
ALB	Albumin
CHO	Cholesterol
TG	Triglycerides
TP	Total protein
IgA	Immunoglobulin A
IgM	Immunoglobulin M
IgG	Immunoglobulin G
ELISA	Enzyme-linked immunosorbent assay
T-AOC	Total antioxidant capacity
CAT	Catalase activity
MDA	Malondialdehyde
T-SOD	Total superoxide dismutase
